# ECOA-3. MLL4, UTX, and EZH2 regulate the expression of transcription factors associated with the epithelial-to-mesenchymal transition of lung cancer during metastasis to the brain at posttranscriptional steps

**DOI:** 10.1093/noajnl/vdab070.003

**Published:** 2021-07-05

**Authors:** Young Zoon Kim

**Affiliations:** Sungkyunkwan University School of Medicine, Changwon, Korea, Republic of

## Abstract

**Purpose:**

The objective of this study was to investigate the epigenetic role of histone lysine methylation/demethylation on the expression of epithelial-to-mesenchymal transition (EMT) associated transcriptional factors (TFs) during the metastasis of lung adenocarcinoma to the brain.

**Methods:**

Paired samples of lung adenocarcinoma and brain metastasis (BM) were analyzed in 46 individual patients. Both samples were obtained by surgical resection or biopsy of the lung and brain. The paraffin-fixed formalin-embedded samples were obtained from the pathology archives in our institute. In samples of lung adenocarcinoma and BM, immunohistochemical staining was performed for epithelial markers, mesenchymal markers, EMT-TFs, histone lysine methyltransferase and demethylase. And the verification of the present result was performed by qRT-PCR.

**Results:**

The immunoreactivity of EMT-TFs such as Slug (15.6% vs. 42.6%, p = 0.005), Twist (23.6% vs. 45.9%, p = 0.010) and ZEB1 (15.0% vs. 55.9%, p = 0.002) was increased in BM compared with that in lung adenocarcinoma. Epigenetic inducers such as H3K4 methyltransferase (MLL4, p = 0.018) and H3K36me3 demethylase (UTX, p = 0.003) were statistically increased, and epigenetic repressors such as EZH2 (H3K27 methyltransferase, p = 0.046) were significantly decreased in BM compared with those in lung adenocarcinoma. The expression of UTX-ZEB1 (R2 linear = 1.204) and MLL4-Slug (R2 linear = 0.987) was increased in direct proportion, and EZH2-Twist (R2 linear = - 2.723) decreased in reverse proportion. The qRT-PCR showed the same results.

**Conclusion:**

The results suggest that certain histone lysine methyltransferase/demethylase, such as MLL4, UTX, and EZH2, regulate the expression of EMT-TFs such as Slug, ZEB1, and Twist epigenetically, which may thereby influence cancer metastasis from the lung to the brain.

